# Beta-blockers provide a differential survival benefit in patients with coronary artery disease undergoing contemporary post-percutaneous coronary intervention management

**DOI:** 10.1038/s41598-020-79214-0

**Published:** 2020-12-17

**Authors:** Pil Hyung Lee, Gyung-Min Park, Seungbong Han, Yong-Giun Kim, Jong-Young Lee, Jae-Hyung Roh, Jae-Hwan Lee, Young-Hak Kim, Seung-Whan Lee

**Affiliations:** 1 Department of Cardiology, Asan Medical Center, University of Ulsan College of Medicine, Seoul, Korea; 2 Department of Cardiology, Ulsan University Hospital, University of Ulsan College of Medicine, 877 Bangeojinsunhwando-ro, Dong-gu, Ulsan, 44033 Korea; 3 Department of Applied Statistics, Gachon University, 1342 Seongnamdaero, Sujeong-gu, Seongnam-si, Gyeonggi-do 13120 Korea; 4 Department of Cardiology, Kangbuk Samsung Hospital, School of Medicine, Sungkyunkwan University, Seoul, Korea; 5 Department of Cardiology, Chungnam National University Sejong Hospital, Sejong, Korea

**Keywords:** Interventional cardiology, Cardiology

## Abstract

Beta-adrenergic receptor blockers are used in patients with coronary artery disease (CAD) to reduce the harmful effects of excessive adrenergic activation on the heart. However, there is limited evidence regarding the benefit of beta-blockers in the context of contemporary management following percutaneous coronary intervention (PCI). We used the nationwide South Korea National Health Insurance database to identify 87,980 patients with a diagnosis of either acute myocardial infarction (AMI; n = 38,246) or angina pectoris (n = 49,734) who underwent PCI between 2013 and 2017, and survived to be discharged from hospital. Beta-blockers were used in a higher proportion of patients with AMI (80.6%) than those with angina (58.9%). Over a median follow-up of 2.2 years (interquartile range 1.2–3.3 years) with the propensity-score matching analysis, the mortality risk was significantly lower in patients treated with a beta-blocker in the AMI group (HR: 0.78; 95% CI 0.69–0.87; *p* < 0.001). However, the mortality risk was comparable regardless of beta-blocker use (HR: 1.07; 95% CI 0.98–1.16; *p* = 0.10) in the angina group. The survival benefit associated with beta-blocker therapy was most significant in the first year after the AMI event.

## Introduction

Excessive catecholamine stimulation increases myocardial oxygen demand and predisposes to the development or worsening of ischemia or atrial and ventricular arrhythmias^1^. Because activation of the adrenergic system beyond the ischemic tolerance occurs in a variety of coronary artery disease (CAD) settings, beta-adrenergic receptor blockers have been evaluated in this patient group to determine the clinical benefits of reducing the adverse effects of sympathetic overactivation on the heart^2^.

Early clinical trials demonstrated that the long-term use of a beta-blocker reduces mortality in patients with acute myocardial infarction (AMI), and these agents, therefore, form the basis of current guidelines, which advocate the beta-blocker use in patients recovering from an AMI regardless of their cardiovascular risk profile^3–5^. However, these recommendations are considered to be out-of-date in contemporary practice where additional therapies (i.e., percutaneous coronary intervention [PCI] with modern versions of drug-eluting stents, antiplatelet agents, and statins) with proven survival benefits are commonly used as part of AMI management. Moreover, the benefits of beta-blockers demonstrated in those AMI trials have been extrapolated to patients with stable CAD who had not experienced a previous myocardial infarction, despite the paucity of evidence from adequately conducted studies^2,6,7^. Therefore, real-world beta-blocker use in patients with CAD has been shown to vary considerably between countries and communities^8^. To address this knowledge gap and to evaluate real-world practice regarding the use of beta-blockers, nationwide cohort data from the National Health Insurance (NHI) database in South Korea have been evaluated, focusing on two representative CAD populations (AMI and angina pectoris) undergoing contemporary post-PCI management.

## Methods

### Data sources

The National Health Insurance (NHI) service of South Korea is a compulsory social insurance service that provides affordable health coverage for all citizens. All healthcare providers are obligated to join the NHI system on a fee-for-service basis^9^. The Health Insurance Review & Assessment (HIRA) service of South Korea is a quasi-governmental organization that systematically evaluates the medical expenses reported from healthcare providers to minimize the risk of redundant and unnecessary medical services. As a result, all NHI claims are reviewed by the HIRA and are systematically classified and recorded in an independent computerized database. Individual diagnoses in the HIRA database are coded according to the International Classification of Diseases, 10th Revision (ICD-10). Information about the drugs, medical devices, and procedures were identified by specific codes from the HIRA. The local institutional review board of the Ulsan University Hospital, Ulsan, Korea approved the study protocol and exempted the requirement for informed consent because the database used for the study consisted of anonymous and de-identified information. This study followed the Strengthening the Reporting of Observational Studies in Epidemiology (STROBE) reporting guideline^10^.

### Study population

The HIRA claims database was used to identify patients aged ≥ 18 years of age who had undergone PCI (M6551, M6552, M6561-4, M6571, and M6572) for the treatment of a CAD (ICD-10 codes I20.X–I25.X) between July 2013 and June 2017. Patients with at least 6 months of eligibility prior to the index day were selected. Patients were excluded if the HIRA database indicated a previous history of CAD (ICD-10 codes I20.X–I25.X) within 6 months of the index day to ensure that the study included only patients with a first diagnosis of CAD. Patients who died during hospitalization after the index procedure were excluded to reduce patient-related confounding factors and to create a more homogeneous beta-blocker-tolerant study population. Patients were categorized into two representative groups: AMI or angina, and the impact of beta-blocker use on patient outcomes was evaluated. AMI was defined using hospital discharge information from the HIRA databases (ICD-10 codes I21.X–I22.X).

### Study variables and endpoint

Individual comorbid conditions were identified using the ICD-10 codes, such as diabetes with or without chronic complications, hyperlipidemia, hypertension, history of heart failure, arrhythmia, valvular heart disease, peripheral vascular disease, cerebrovascular disease, chronic pulmonary disease, moderate-to-severe liver disease, renal disease, cancer, and rheumatologic disease (Table S1). Patients were also considered to have diabetes mellitus, hypertension, and hyperlipidemia if anti-diabetic, anti-hypertensive, and anti-hyperlipidemic drugs were identified from the medication codes in the HIRA database within 6 months of the index day. The Charlson comorbidity index was calculated from these data to measure the patients’ comprehensive life expectancy^11^.

Cardiovascular medication was characterized as antiplatelet agents, statins, angiotensin-converting enzyme inhibitors, angiotensin II receptor antagonists, and beta-blockers. Patients were grouped into mutually exclusive exposure categories according to the use of beta-blockers (i.e., beta-blocker group or no beta-blocker group) at the time of hospital discharge. Specific subtypes of beta-blockers and stents used during PCI (drug-eluting stent: J5083XXX or J8083XXXX; bioresorbable vascular scaffold: J5084XXXX; bare-metal stent: J5231XXX, J5232XXX, or J8231XXXX) were identified for each patient. Newer-generation drug-eluting stents were exclusively used in the Korean market during the study period. Non-stent coronary balloon angioplasty was assigned if device codes were not accompanied by a code indicating a drug-eluting stent, bioresorbable vascular scaffold, or a bare-metal stent.

The primary endpoint of this study was all-cause mortality. Death was identified by all in- and outpatient claims records that indicated death. All claims data until December 2017 were used.

### Statistical analysis

Statistical analyses of beta-blocker vs. no beta-blocker groups were conducted separately for AMI and angina group. Summary statistics for continuous and categorical variables are presented by mean ± standard deviation or frequency (percentage, %). Continuous variables were compared using the Wilcoxon rank-sum test, while categorical variables were compared using the χ^2^ statistics. Cumulative incidence rates of all-cause death were computed by the product limit estimator. We also compared cumulative incidence rates between the beta-blocker and no beta-blocker groups using the log-rank test. Cox regression analysis was employed to find any association between beta-blocker usage and all-cause mortality. The proportionality assumptions were verified by the Shoenfeld residual test. To control any potential confounding factors, we used the propensity-score matching analysis based on variables such as age, gender, comorbidities, type and number of stents, Charlson comorbidity score, and cardiovascular medications. Propensity scores were estimated nonparametrically using “MatchIt” function in the R package “MatchIt”. The MatchIt function does not assume any parametric relationship between the beta-blocker variable and the confounding variables via a machine learning algorithm. Matching was conducted by a 1:1 nearest neighbor random matching with caliper size 0.05 multiplied by the standard deviation for linearly transformed propensity scores (logit-transformation). The quality of matching was evaluated by computing the standardized difference in means for the two groups before and after matching (Fig. S1). Wilcoxon signed-rank test or the McNemar test was used to assess the covariate balance between the two matched groups. Cox proportional hazards regression model with robust standard errors that accounts for the clustering of the pairs was used to compare the risks of outcomes in the matched cohort. The *P*-values were two-sided and those < 0.05 were considered significant. Data analyses were performed using R software version 3.6.1 (R Foundation for Statistical Computing, Vienna, Austria).

## Results

### Study population and characteristics

**Figure 1:**
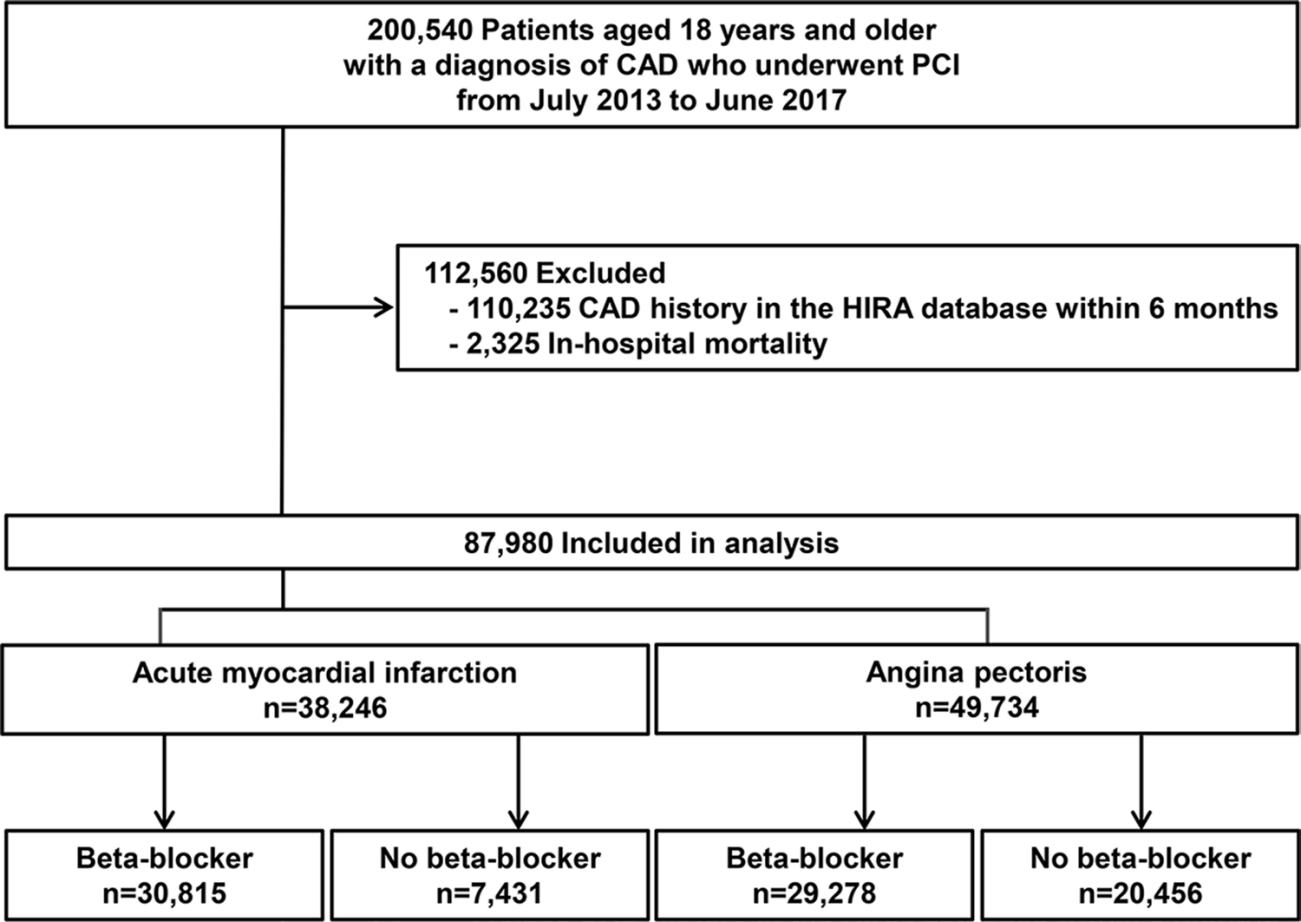
Overview of the study population. CAD, coronary artery disease; HIRA, Health Insurance Review and Assessment; PCI, percutaneous coronary intervention.

In the study period, a total of 200,540 patients aged ≥ 18 years were diagnosed with CAD and underwent PCI. Of these, 87,980 patients met the eligibility criteria; 60,093 of which (68.3%) were treated with a beta-blocker. A total of 38,246 patients had been diagnosed with AMI, and 49,734 patients were diagnosed with angina as the first event of CAD (Fig. 1). The overall baseline patient characteristics are presented in Table 1. The mean age of the cohort was 64.4 years and 71.4% were male. Overall, diabetes was observed in 29,814 patients (33.9%), and 1749 (2.0%) suffered from malignancy. When compared with patients with AMI, those with angina were generally older and had a higher frequency of cardiovascular risk factors. The majority of the study population was treated with drug-eluting stents (93.6%); the average number of stents used was 1.4 ± 0.7. After PCI, secondary preventive drugs were used routinely, including aspirin (93.8%), P2Y12 receptor inhibitors (99.1%), and statins (91.7%).

**Table 1:** Baseline characteristics of the study population.

Characteristics	Overall n = 87,980	AMI n = 38,246	Angina n = 49,734
Age, years	65 (56–74)	63 (54–74)	66 (57–74)
Male	62,776 (71.4)	29,172 (76.3)	33,604 (67.6)
Comorbid conditions			
Diabetes	29,814 (33.9)	10,416 (27.2)	19,398 (39.0)
Hyperlipidemia	37,108 (42.2)	11,365 (29.7)	25,743 (51.8)
Hypertension	51,986 (59.1)	18,428 (48.2)	33,558 (67.5)
History of heart failure	5,538 (6.3)	1,297 (3.4)	4,241 (8.5)
Cardiac arrhythmia	5,768 (6.6)	1,254 (3.3)	4,514 (9.1)
Valvular heart disease	284 (0.3)	60 (0.2)	224 (0.5)
Peripheral vascular disorder	9,926 (11.3)	3,318 (8.7)	6,608 (13.3)
Cerebrovascular disease	10,605 (12.1)	3,153 (8.2)	7,452 (15.0)
Chronic pulmonary disease	11,838 (13.5)	4,186 (10.9)	7,652 (15.4)
Moderate-to-severe liver disease	34 (0.04)	13 (0.03)	21 (0.04)
Renal disease	4,089 (4.6)	1,163 (3.0)	2,926 (5.9)
Malignancy	1,749 (2.0)	642 (1.7)	1,107 (2.2)
Rheumatic disease	143 (0.2)	49 (0.1)	94 (0.2)
Charlson comorbidity index	1 (0–2)	1 (0–2)	1 (0–2)
Type of treatment for PCI			
Drug-eluting stent	82,310 (93.6)	36,011 (94.2)	46,299 (93.1)
Bioresorbable vascular scaffold	568 (0.6)	250 (0.7)	318 (0.6)
Bare-metal stent	599 (0.7)	276 (0.7)	323 (0.6)
Plain balloon angioplasty	4,503 (5.1)	1,709 (4.5)	2,794 (5.6)
Number of stents per person	1 (1–2)	1 (1–2)	1 (1–2)
Medication at discharge			
Aspirin	82,567 (93.8)	36,067 (94.3)	46,500 (93.5)
P2Y12 receptor antagonists	87,194 (99.1)	37,107 (99.6)	49,087 (98.7)
Statins	80,669 (91.7)	36,324 (95.0)	44,345 (89.2)
ACEI or ARBs	56,389 (64.1)	27,264 (71.3)	29,125 (58.6)

Data are shown as the median (interquartile range) or n (%).

*ACEI* angiotensin-converting enzyme inhibitor, *AMI* acute myocardial infarction, *ARB* angiotensin II receptor antagonist, *PCI* percutaneous coronary intervention.

### Beta-blocker use

Beta-blockers were used in a higher proportion of patients with AMI (80.6%) than those with angina (58.9%). Carvedilol (36.6%) and bisoprolol (25.1%) were the most commonly prescribed beta-blockers, followed by nebivolol (7.0%) and propranolol (3.2%); these prescription patterns were similar in both the AMI and angina groups (Table S2). Table 2 shows the patient characteristics according to the beta-blocker use in each of the diagnosis categories. Overall, patients who did not receive beta-blocker tended to be older and had a higher prevalence of peripheral or cerebrovascular disease. However, differences in patients characteristics between the beta-blocker versus no beta-blocker groups were also present according to the diagnostic category, i.e., patients who received a beta-blocker for angina were more likely to be female and have a history of heart failure or renal disease, whereas those who received beta-blockers following an AMI were less likely to be female or have diabetes, heart failure, or renal disease. The Charlson comorbidity index score was higher in patients receiving no beta-blockers in the AMI group, but was similar between beta-blocker and no beta-blocker group in the angina group. The proportion of patients treated with beta-blockers during the study period is shown in Fig. S2. Beta-blocker use was consistently high after AMI (~ 80%) throughout the 4 years study period. However, the use of beta-blockers in the angina group (approximately 60%) gradually decreased over time.

**Table 2:** Characteristics of the study patients according to beta-blocker use.

Characteristics	AMI n = 38,246			Angina n = 49,734		
	No beta-blocker n = 7431	Beta-blocker n = 30,815	*P*-value	No beta-blocker n = 20,456	Beta-blocker n = 29,278	*P*-value
Baseline characteristics						
Age, years	65 (55–75)	62 (53–73)	< 0.001	66 (58–74)	66 (57–74)	0.005
Male	5,574 (75.0)	23,598 (76.6)	0.005	14,051 (68.7)	19,553 (66.8)	< 0.001
Diabetes	2,105 (28.3)	8,311 (27.0)	0.019	8,008 (39.1)	11,390 (38.9)	0.581
Hyperlipidemia	2,298 (30.9)	9,067 (29.4)	0.011	11,166 (54.6)	14,577 (49.8)	< 0.001
Hypertension	3,682 (49.5)	14,746 (47.9)	0.009	13,841 (67.7)	19,717 (67.3)	0.460
History of heart failure	303 (4.1)	994 (3.2)	< 0.001	1,422 (7.0)	2,819 (9.6)	< 0.001
Cardiac arrhythmia	245 (3.3)	1,009 (3.3)	0.913	1,774 (8.7)	2,740 (9.4)	0.009
Valvular heart disease	20 (0.3)	40 (0.1)	0.013	94 (0.5)	130 (0.4)	0.838
Peripheral vascular disease	661 (8.9)	2,657 (8.6)	0.449	2,790 (13.6)	3,818 (13.0)	0.053
Cerebrovascular disease	709 (9.5)	2,444 (7.9)	< 0.001	3,156 (15.4)	4,296 (14.7)	0.021
Chronic pulmonary disease	918 (12.4)	3,268 (10.6)	< 0.001	3,152 (15.4)	4,500 (15.4)	0.910
Moderate-to-severe liver disease	3 (0.04)	10 (0.03)	0.726	6 (0.03)	15 (0.05)	0.275
Renal disease	268 (3.6)	895 (2.9)	0.002	1,119 (5.5)	1,807 (6.2)	0.001
Malignancy	157 (2.1)	485 (1.6)	0.002	479 (2.3)	628 (2.1)	0.147
Rheumatologic disease	10 (0.1)	39 (0.1)	0.857	39 (0.2)	55 (0.2)	0.999
Charlson comorbidity index	1 (0–2)	1 (0–1)	< 0.001	1 (0–2)	1 (0–2)	0.481
Type of treatment for PCI			< 0.001			< 0.001
Drug-eluting stent	6,856 (92.3)	29,155 (94.6)		18,969 (92.7)	27,330 (93.3)	
Bioresorbable vascular scaffold	38 (0.5)	212 (0.7)		166 (0.8)	152 (0.5)	
Bare-metal stent	41 (0.6)	235 (0.8)		135 (0.7)	188 (0.6)	
Plain balloon angioplasty	496 (6.7)	1,213 (3.9)		1,186 (5.8)	1,608 (5.5)	
Number of stents per person	1 (1–2)	1 (1–2)	0.753	1 (1–2)	1 (1–2)	< 0.001
Medication at discharge						
Aspirin	6,796 (91.5)	29,271 (95.0)	< 0.001	18,829 (92.0)	27,671 (94.5)	< 0.001
P2Y12 receptor antagonists	7,366 (99.1)	30,741 (99.8)	< 0.001	4,964 (98.4)	25,269 (99.6)	< 0.001
Statins	6,740 (90.7)	29,584 (96.0)	< 0.001	20,032 (97.9)	29,055 (99.2)	< 0.001
ACEI or ARBs	3,734 (50.2)	23,530 (76.4)	< 0.001	9,069 (44.3)	20,056 (68.5)	< 0.001

Data are shown as the median (interquartile range) or n (%).

*ACEI* angiotensin-converting enzyme inhibitor, *AMI* acute myocardial infarction, *ARB* angiotensin II receptor antagonist, *PCI* percutaneous coronary intervention.

### Clinical outcomes

**Figure 2:**
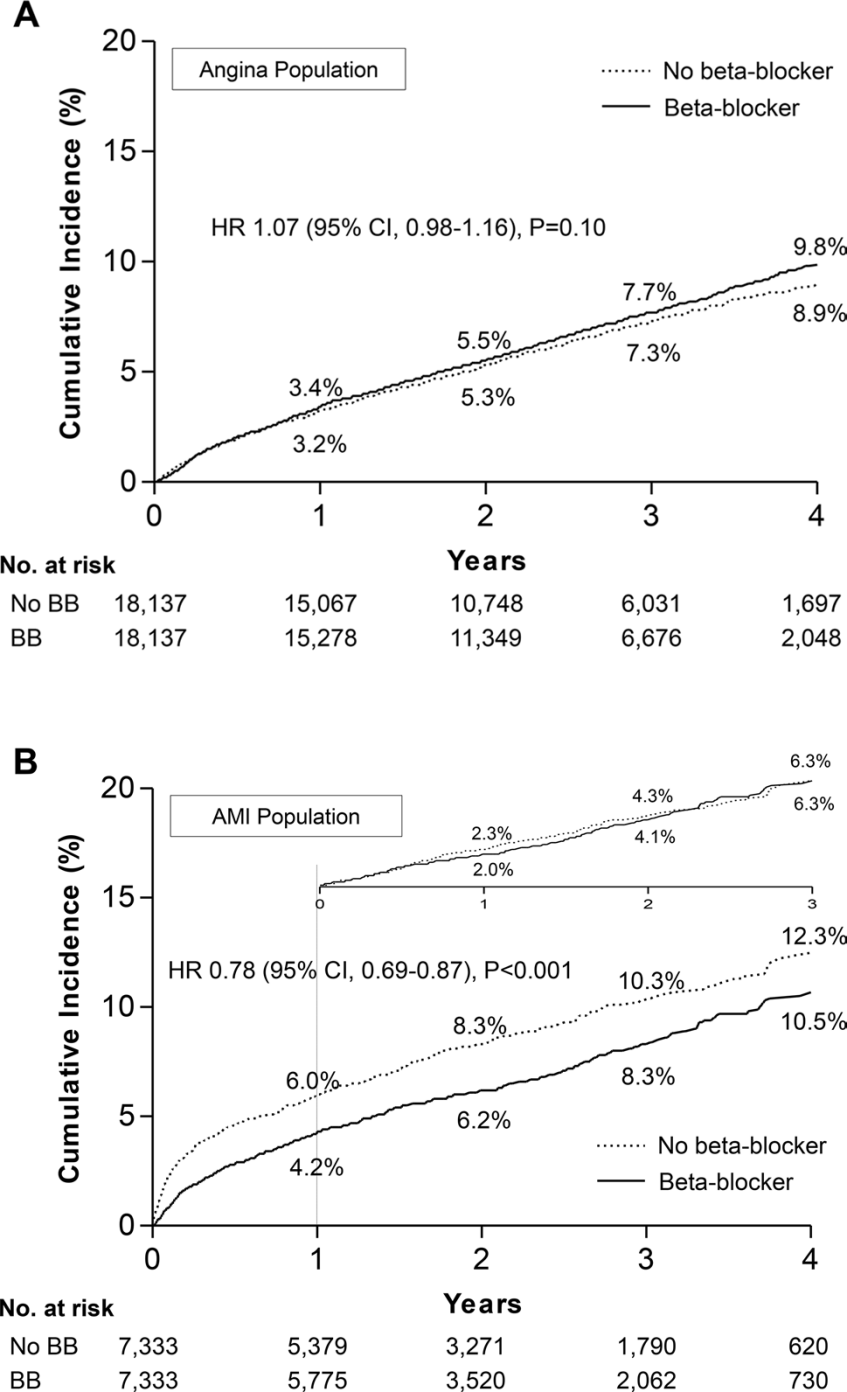
Kaplan–Meier cumulative event curves for mortality in the matched cohort. The cumulative incidence rates for all-cause death between the beta-blocker and no beta-blocker therapy groups in patients with AMI (**a**) and those with angina (**b**). The numbers in each figure represent the cumulative incidence rates at each time point. AMI, acute myocardial infarction; BB, beta-blocker.

The median length of follow-up was 2.2 years (interquartile range, 1.2–3.3 years). The primary outcome of death occurred in 3748 (6.2%) patients in the beta-blocker group and 1845 patients (6.6%) in the no beta-blocker group. Overall, the mortality rate was significantly lower in patients treated with a beta-blocker compared with those without (2 year event rate: 5.5% vs. 6.1%; log-rank *p* = 0.003) (Fig. S3). After propensity-score matching to assemble a cohort of patients with clinical equipoise for beta-blocker and no beta-blocker therapy at baseline, there were 7333 matched pairs of patients in the AMI cohort and 18,137 pairs in the angina cohort. Baseline characteristics in the propensity-score matched cohort are shown in Table S3, and the event rates and risks for clinical outcomes of the matched cohort are shown in Fig. 2. A differential prognosis was found between the two populations in that there was no difference in the risk of death between the beta-blocker and no beta-blocker groups in patients with angina (hazard ratio [HR]: 1.07; 95% confidence interval [CI]: 0.98–1.16; *p* = 0.10) (Fig. 2a), whereas the mortality risk was significantly lower with beta-blocker treatment in patients with AMI (HR: 0.78; 95% CI 0.69–0.87; *p* < 0.001) (Fig. 2b). The survival benefit associated with beta-blocker use was significant within 1 year (HR: 0.81; 95% CI 0.70–0.94; *p* = 0.005) of the AMI event, but not thereafter (HR: 0.94; 95% CI 0.78–1.15; *p* = 0.60). The treatment effect for the primary outcome in prespecified subgroups of the matched AMI cohort is shown in Fig. S4. The propensity of mortality risk between beta-blocker and no beta-blocker treatment across the subgroups was generally consistent with the overall results of AMI.

## Discussion

This nationwide cohort study included data from 87,980 patients with a first diagnosis of AMI or angina who underwent PCI and received contemporary medical treatment in Korea. The main findings are as follows: (1) beta-blockers were prescribed in a high percentage of patients after AMI from 2013–2017 in real-world clinical practice; (2) treatment with beta-blockers was associated with a significant reduction in mortality in patients with AMI but not in those with angina; (3) the survival benefit associated with beta-blocker use was most significant within 1 year of the AMI event.

No placebo-controlled trials have been conducted to evaluate the effect of beta-blockers on clinical outcomes in patients with stable CAD^12^. Our study result of the angina cohort is consistent with observational studies that have questioned the benefit of beta-blockers on survival in such a clinical scenario. In the REACH registry and a post hoc analysis of the CHARISMA trial, beta-blockers were not associated with a lower risk of all-cause or cardiovascular death in established CAD patients regardless of a history of myocardial infarction^13,14^. In the more recent international CLARIFY registry, beta-blocker use was not associated with lower 5-year mortality among 22,006 established CAD patients who have not experienced myocardial infarction or revascularization within the previous 3 months of enrollment^15^. Of note, our study included a relatively homogeneous population with a first diagnosis of CAD without prior myocardial infarction by study design. Thus, the findings in our angina cohort may be somewhat expected because patients are highly likely to have a normal left ventricular function and were followed-up after receiving PCI for a given culprit coronary lesion. Nevertheless, considering that outcomes may differ between studies according to the patient profiles or inclusion criteria, our study add to the contemporary evidence that the use of beta-blockers is not mandated on the prognostic ground in CAD patients without evidence of myocardial injury who undergoes PCI.

Available evidence strongly supports the use of beta-blockers to reduce mortality in AMI patients with left ventricular ejection fraction ≤ 40%. In a population of patients with left ventricular dysfunction post-myocardial infarction, the CAPRICORN study demonstrated that over a mean period of 1.3 years, carvedilol reduced the risk of mortality by 23% compared with placebo^3^. Together with the unequivocal survival benefit of beta-blockers in patients with heart failure with reduced ejection fraction^16–21^, there is no doubt that considering beta-blockers for these selected AMI patients is appropriate. However, there is continued debate about the broader use of beta-blockers in unselected patients after AMI^22–25^. A considerable number of studies have shown a consistent mortality benefit associated with beta-blocker use in these patients, with study periods extending from several months to up to 3 years^4^. However, these trials were primarily conducted in the 1980s and 1990s, before the widespread use of PCI and other proven medical therapies, raising the question of whether the demonstrated benefits would still be observed in the context of contemporary clinical management. The lack of study data is a challenge that is faced in real-world practice. The current study, based on reliable nationwide data that included all CAD patients who underwent PCI from 2013–2017, was designed to provide valuable insight and evidence to inform daily clinical practice.

The essential finding of our study is that in the AMI setting, beta-blocker use was associated with an approximately 25% and 15% reduction in mortality at 2 and 4 years, respectively. This result should be considered in light of the fact that the majority of the study population received modern PCI using drug-eluting stents and secondary preventive drugs, including antiplatelet agents and statins. These modern interventional advances and preventive drugs have resulted in a decline in the mortality rate of patients with AMI compared with early periods, and this was presumed to be the reason why the survival benefit associated with beta-blocker use that was observed in historical trials was not consistently reproduced in recent observational studies and meta-analyses^22^. However, because the mechanism by which reperfusion therapy, platelet inhibitors, or statins improves clinical outcomes differs from that of beta-blockers, it is reasonable to assume that the benefits of beta-blocker therapy will be preserved in patients receiving contemporary post-myocardial infarction management. One important observation in our analysis is that the survival benefit associated with beta-blocker use was most significant within 1 year of an AMI event. Our findings are in broad agreement with the nationwide French registry, which included 2697 patients with AMI and without left ventricular dysfunction, in that early beta-blocker use was associated with lower 30-day mortality, but their discontinuation at 1 year was not associated with 5-year mortality^26^. This implies that patients who survived during hospitalization after PCI for AMI remain at risk of major cardiac events due to vulnerable myocardium and coronary vessels, mostly at the early periods after AMI, during which beta-blockers can be of benefit. To date, the utility of prolonged beta-blocker treatment after AMI is questionable and should be a subject of future investigation.

The results reported here support the need for contemporary randomized trials that examine the usefulness and appropriate duration of beta-blockers in a broad AMI population. The lack of recent evidence to support the routine use of beta-blockers in AMI patients who have undergone PCI has resulted in contradictory recommendations among the guidelines. The American Heart Association guidelines recommend oral beta-blockers as a class I indication for all patients with AMI for at least 3 years^5^, whereas the European guidelines provide no recommendations for beta-blockers during and after an AMI episode in patients with normal or mildly depressed left ventricular function^6,7^. As a result, the use of beta-blockers differs substantially among cardiovascular societies, from less than half of eligible patients to > 80%^27–32^. Although a high proportion of AMI patients in the current study received beta-blockers, it is interesting to note that the prescription rate rose sharply since 2013 due to the government’s adequacy evaluation project for ischemic heart disease in Korea^33^. Two randomized trials, evaluating the effect of beta-blocker therapy after AMI in patients without left ventricular dysfunction, are in progress and will provide further guidance on these issues^34,35^.

This study has several limitations. First, the retrospective and observational design is associated with inherent bias. Second, similar to previous studies using an administrative database, clinical data regarding the cardiac test findings or vital signs of each individual were not available, further limiting the adjustment of meaningful clinical factors. Importantly, the lack of left ventricular ejection fraction information hindered the evaluation of the effectiveness of beta-blockers in subgroups with or without left ventricular dysfunction within each study group. Third, in the same context, it would be difficult to interpret the finding that beta-blocker treatment was associated with a neutral risk of mortality in patients with a history of heart failure shown in Fig. S4. A prior diagnosis of heart failure is presumed to be a non-ischemic etiology and encompasses both reduced and preserved left ventricular ejection fraction, and an absence of a history of heart failure does not necessarily mean that the patient had preserved left ventricular systolic function after the index PCI. Fourth, drug use was determined only by the discharge medication, and full information on the drug adherence was unavailable as other retrospective studies. Finally, as this study only included a Korean population, it is uncertain whether these findings can be applied to other ethnic groups with different patient characteristics and procedural approaches.

In summary, in this population of unselected CAD patients who underwent contemporary post-PCI management, beta-blocker treatment was associated with a significant reduction in mortality in patients with AMI but not in those with angina. These results support the current guidelines, which recommend beta-blockers as first-line therapy to improve prognosis after AMI and to improve ischemic symptoms in patients with stable CAD. The findings should be confirmed by randomized clinical trials with an appropriate duration of follow-up.

## Supplementary information

Supplementary Information.

## Data Availability

The present study analyzed the National Health Insurance (NHI) claims data in South Korea. Data of the NHI claims are accessible to researchers after permission of the Health Insurance Review & Assessment Service (HIRA) in South Korea. Qualified, interested researchers may request access to these data from the HIRA (http://opendata.hira.or.kr/home.do).
