# Supplementary material for: Beta-blockers provide a differential survival benefit in patients with coronary artery disease undergoing contemporary post-percutaneous coronary intervention management
Source: Sci Rep. 2020 Dec 17;10:22121. doi: 10.1038/s41598-020-79214-0 (PMC7746699; doi:10.1038/s41598-020-79214-0)
Supplement: Supplementary file 1 — Supplementary Information. [file 41598_2020_79214_MOESM1_ESM.docx]

**Supplementary Information**

Supplement to: **Beta-blockers provide a differential survival benefit in patients with coronary artery disease undergoing contemporary post-percutaneous coronary intervention management**

**Investigators:** Pil Hyung Lee, Gyung-Min Park, Seungbong Han, Yong-Giun Kim, Jong-Young Lee, Jae-Hyung Roh, Jae-Hwan Lee, Young-Hak Kim, Seung-Whan Lee

| Contents | Page |
| --- | --- |
| I. Supplemental Tables |  |
| A. Table 1. Definition of covariates | 3 |
| B. Table 2. Type of beta-blocker used | 4 |
| C. Table 3. Characteristics of propensity-score matched patients according to the beta-blocker use | 5 |

II. Supplemental Figures

| A. Fig. 1. Covariate balance before and after matching for each comparison | 7 |
| --- | --- |
| B. Fig. 2. Trends in beta-blocker use  C. Fig. 3. Kaplan-Meier cumulative event curves for mortality in the unmatched cohort  D. Fig. 4. Subgroup analysis for the primary outcome in the matched AMI cohort | 8  9  11 |

**I. Supplemental Tables**

***Supplemental Table 1:* Definition of covariates**

| **Diagnosis** | **Definition** |
| --- | --- |
| Diabetes mellitus | E10.0, E10.1, E10.6, E10.8–E11.1, E11.6, E11.8–E12.1, E12.6, E12.8–E13.1, E13.6, E13.8–E14.1, E14.6, E14.8, E14.9, E10.2–E10.5, E10.7, E11.2–E11.5, E11.7, E12.2–E12.5, E12.7, E13.2–E13.5, E13.7, E14.2–E14.5, E14.7 OR a minimum of one prescription of anti-diabetic drugs (sulfonylureas, metformin, α-glucosidase inhibitors, thiazolidinediones, meglitinides, dipeptidyl peptidase-4 inhibitors, sodium-glucose cotransporter 2 inhibitors, glucagon like peptide-1 receptor agonists, and insulins) |
| Hyperlipidemia | E78.0–E78.5 OR a minimum of one prescription of anti-hyperlipidemic drugs (statins, ezetimibe, fibric acid derivatives, omega-3 fatty acids, and nicotinic acid) |
| Hypertension | I10.x, I11.x–I13.x, I15.x OR a minimum of one prescription of anti-hypertensive drugs (angiotensin-converting enzyme inhibitors or Angiotensin II receptor blockers, calcium-channel blockers, β-blockers, and thiazide type diuretics) |
| History of heart failure | I09.9, I11.0, I13.0, I13.2, I25.5, I42.0, I42.5–I42.9, I43.x, I50.x, P29.0 |
| Cardiac arrhythmia | I44.1–I44.3, I45.6, I45.9, I47.x–I49.x, R00.0, R00.1, R00.8, T82.1, Z45.0, Z95.0 |
| Valvular heart disease | A52.0, I05.x-I08.x, I09.1, I09.8, I34.x–I39.x, Q23.0–Q23.3, Z95.2–Z95.4 |
| Peripheral vascular disorder | I70.x, I71.x, I73.1, I73.8, I73.9, I77.1, I79.0, I79.2, K55.1. K55.8, K55.9, Z95.8, Z95.9 |
| Cerebrovascular disease | G45.x, G46.x, H34.0, I60.x–169.x |
| Chronic pulmonary disease | I27.8, I27.9, J40.x–J47.x, J60.x–J67.x, J68.4, J70.1, J70.3 |
| Moderate-to-severe liver disease | I85.0, I85.9, I86.4, I98.2, K70.4, K71.1, K72.1, K72.9, K76.5, K76.6, K76.7 |
| Renal disease | I12.0, I13.1, N03.2–N03.7, N05.2–N05.7, N18.x, N19.x, N25.0, Z49.0–Z49.2, Z94.0, Z99.2 |
| Malignancy | C00.x–C97.x |
| Rheumatic disease | M05.x, M06.x, M31.5, M32.x–M34.x, M35.1, M35.3, M36.0 |

***Supplemental Table 2:* Type of beta-blocker used**

| **Drugs** | **Overall**  **n=87,980** | **AMI**  **n=38,246** | **Angina**  **n=49,734** |
| --- | --- | --- | --- |
| Carvedilol | 32,179 | 17,497 | 14,682 |
| Bisoprolol | 22,094 | 11,316 | 10,778 |
| Nebivolol | 6,169 | 3,162 | 3,007 |
| Propranolol | 2,828 | 1,169 | 1,659 |
| Atenolol | 1,245 | 341 | 904 |
| Metoprolol | 1,077 | 373 | 704 |
| Bevantolol | 142 | 51 | 91 |
| Celiprolol | 129 | 42 | 87 |
| Amosulalol | 71 | 14 | 57 |
| Betaxolol | 60 | 8 | 52 |
| Sotalol | 38 | 5 | 33 |
| Arotinolol | 27 | 7 | 20 |
| Beta-blocker was changed to a different beta-blocker during hospitalization | 5,696 | 3,036 | 2,660 |

AMI, acute myocardial infarction

***Supplemental Table 3:* Characteristics of propensity-score matched patients according to the beta-blocker use**

| **Characteristics** | **AMI**  **n=14,666** | | | **Angina**  **n=36,274** | | |
| --- | --- | --- | --- | --- | --- | --- |
|  | **No beta-blocker n=7,333** | **Beta-blocker n=7,333** | **P-value** | **No beta-blocker n=18,137** | **Beta-blocker n=18,137** | **P-value** |
| Baseline characteristics |  |  |  |  |  |  |
| Age, years | 65 (55–75) | 65 (55–75) | 0.632 | 66 (57–74) | 66 (57–74) | 0.418 |
| Male | 5,513 (75.2) | 5,518 (75.2) | 0.245 | 12,345 (68.1) | 12,295 (67.8) | 0.653 |
| Diabetes | 2,070 (28.2) | 2,131 (29.1) | 0.610 | 6,971 (38.4) | 7,057 (38.9) | 0.939 |
| Hyperlipidemia | 2,264 (30.9) | 2,260 (30.8) | 0.625 | 9,527 (52.5) | 9,627 (53.1) | 0.903 |
| Hypertension | 3,624 (49.4) | 3,591 (49.0) | 0.426 | 12,195 (67.2) | 12,344 (68.1) | 0.246 |
| History of heart failure | 290 (4.0) | 289 (3.9) | 0.103 | 1,351 (7.4) | 1,434 (7.9) | 0.618 |
| Arrhythmia | 243 (3.3) | 242 (3.3) | 0.779 | 1,608 (8.9) | 1,623 (8.9) | 0.911 |
| Valvular disease | 18 (0.2) | 18 (0.2) | 0.243 | 75 (0.4) | 76 (0.4) | 0.871 |
| Peripheral vascular disease | 651 (8.9) | 638 (8.7) | 0.289 | 2,406 (13.3) | 2,443 (13.5) | 0.779 |
| Cerebrovascular disease | 689 (9.4) | 726 (9.9) | 0.352 | 2,726 (15.0) | 2,729 (15.0) | 0.065 |
| Chronic pulmonary disease | 888 (12.1) | 860 (11.7) | 0.854 | 2,782 (15.3) | 2,758 (15.2) | 0.077 |
| Moderate-to-severe liver disease | 3 (0.04) | 2 (0.03) | 0.371 | 6 (0.03) | 5 (0.03) | 0.070 |
| Renal disease | 259 (3.5) | 237 (3.2) | 0.428 | 992 (5.5) | 1,013 (5.6) | 0.177 |
| Cancer | 143 (2.0) | 144 (2.0) | 0.148 | 402 (2.2) | 411 (2.3) | 0.356 |
| Rheumatologic disease | 10 (0.2) | 9 (0.1) | 0.999 | 33 (0.2) | 34 (0.2) | 0.464 |
| Charlson comorbidity index | 1 (0–2) | 1 (0–2) | 0.280 | 1 (0–2) | 1 (0–2) | 0.079 |
| Type of treatment for PCI |  |  |  |  |  |  |
| Drug-eluting stent | 6,806 (92.8) | 6,818 (93.0) | 0.914 | 16,901 (93.2) | 16,894 (93.1) | 0.157 |
| Bioresorbable vascular scaffold | 38 (0.5) | 41 (0.6) | 0.999 | 117 (0.6) | 112 (0.6) | 0.999 |
| Bare-metal stent | 40 (0.5) | 50 (0.7) | 0.343 | 114 (0.6) | 112 (0.6) | 0.999 |
| Number of stent per person | 1 (1–2) | 1 (1–2) | 0.261 | 1 (1–2) | 1 (1–2) | 0.056 |
| Medication at discharge |  |  |  |  |  |  |
| Antiplatelet agents | 7,325 (99.9) | 7,320 (99.8) | 0.228 | 18,064 (99.6) | 18,060 (99.6) | 0.360 |
| Statin | 6,735 (91.8) | 6,722 (91.7) | 0.153 | 16,311 (89.9) | 16,250 (89.6) | 0.440 |
| ACEI/ARB | 3,734 (50.9) | 3,799 (51.8) | 0.526 | 9,062 (50.0) | 9,117 (50.3) | 0.124 |

Data are expressed as n (%) and median (interquartile range).

ACEI, angiotensin-converting enzyme inhibitor; AMI, acute myocardial infarction; ARB, angiotensin receptor blocker; PCI; percutaneous coronary intervention

**I. Supplemental Figures**

***Supplemental Fig. 1:* Covariate balance before and after matching for each comparison**

| **A**  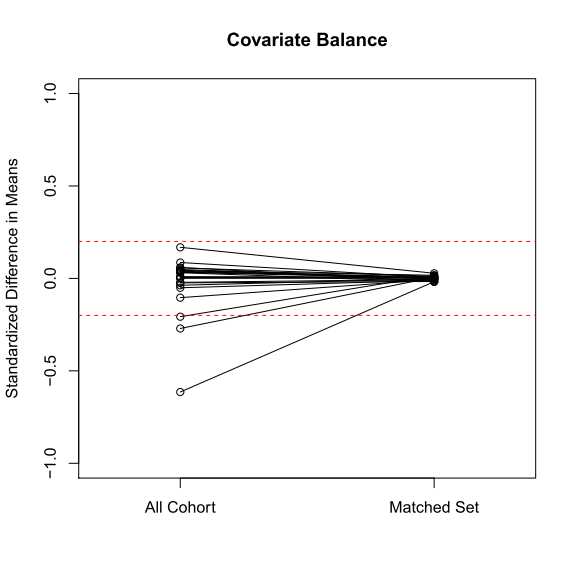 | **B**  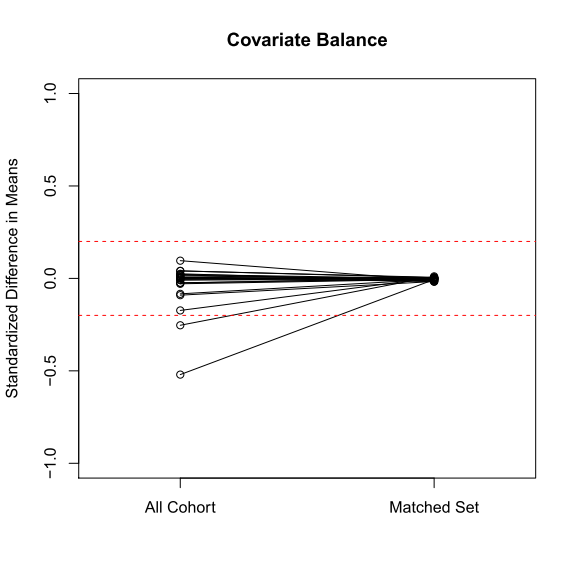 |
| --- | --- |

A; Acute myocardial infarction population, B; Angina pectoris population

***Supplemental Fig. 2:* Trends in beta-blocker use**


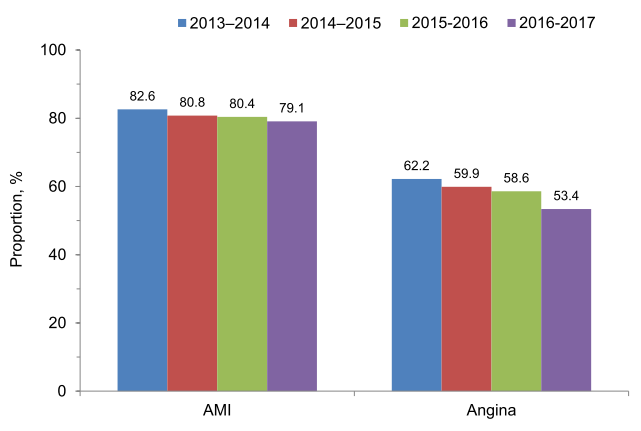


AMI, acute myocardial infarction

***Supplemental Fig. 3:* Kaplan-Meier cumulative event curves for mortality in the unmatched cohort**

| **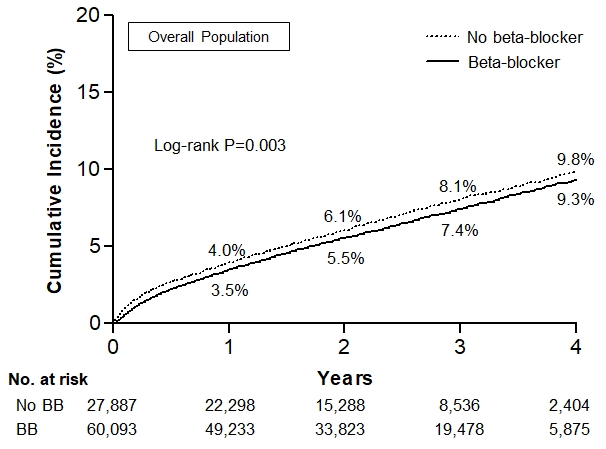**  **A** |
| --- |
| **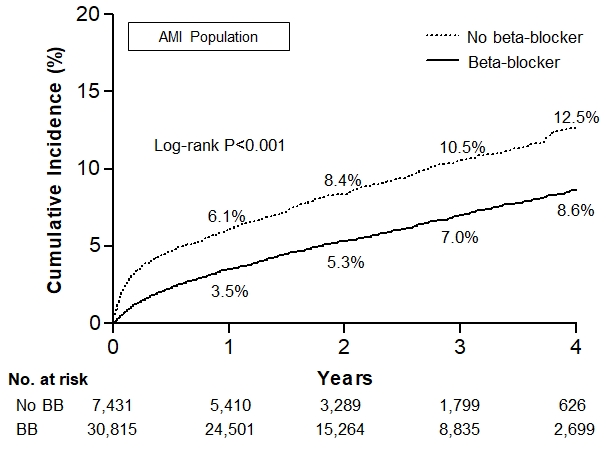**  **B** |
| **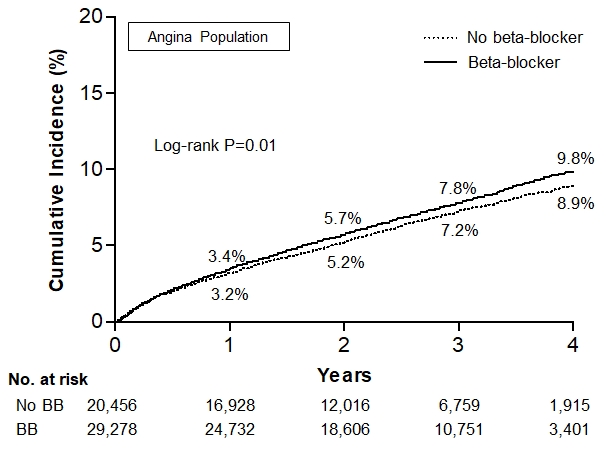**  **C** |

The cumulative incidence rates for all-cause death between the beta-blocker and no beta-blocker therapy groups in the overall population (A), patients with AMI (B), and patients with angina (C).

The numbers in each figure represent the cumulative incidence rates at each time point.

AMI, acute myocardial infarction; BB, beta-blocker

***Supplemental Fig. 4:* Subgroup analysis for the primary outcome in the matched AMI cohort**


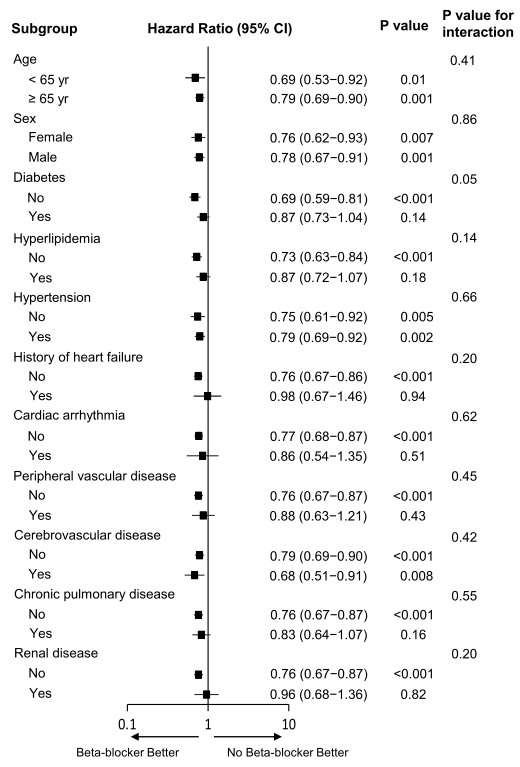


Hazard ratios are for the beta-blocker group compared with the no beta-blocker group. The P-value for interaction represents the likelihood of interaction between the subgroups and the treatment.

AMI, acute myocardial infarction; CI, confidence interval
